# Genetic Association of Hepatitis C-Related Mixed Cryoglobulinemia: A 10-Year Prospective Study of Asians Treated with Antivirals

**DOI:** 10.3390/v13030464

**Published:** 2021-03-11

**Authors:** Ming-Ling Chang, Su-Wei Chang, Shiang-Chi Chen, Rong-Nan Chien, Chia-Lin Hsu, Ming-Yu Chang, Cathy S. J. Fann

**Affiliations:** 1 Division of Hepatology, Department of Gastroenterology and Hepatology, Chang Gung Memorial Hospital, Taoyuan 333423, Taiwan; minglingc@cloud.cgmh.org.tw; 2 Department of Medicine, College of Medicine, Chang Gung University, Taoyuan 333423, Taiwan; p123073@gmail.com; 3 Clinical Informatics and Medical Statistics Research Center, Chang Gung University, Taoyua 333423, Taiwan; shwchang@mail.cgu.edu.tw; 4 Division of Allergy, Asthma, and Rheumatology, Department of Paediatrics, Chang Gung Memorial Hospital, Taoyuan 333423, Taiwan; 5 Department of Nursing, Taipei Medical University, Taipei 11031, Taiwan; rossie7766@gmail.com; 6 Institute of Biomedical Sciences, Academia Sinica, Taipei 115024, Taiwan; chia.lin@ibms.sinica.edu.tw; 7 Division of Pediatric Neurologic Medicine, Chang Gung Children’s Hospital, Taoyuan 333423, Taiwan; 8 Division of Pediatrics, Chang Gung Memorial Hospital, Keelung 20401, Taiwan

**Keywords:** HCV, mixed cryoglobulinemia, IFNL3, ARNTL, PAI-1

## Abstract

Genetic profiles of hepatitis C virus (HCV)-associated mixed cryoglobulinemia (MC) in Asians remain elusive. A 10-year prospective cohort study was conducted with 1043 consecutive HCV Ab-positive Taiwanese surveyed with 13 single nucleotide polymorphisms (SNPs). Of 1043, 589 (56.5%) had baseline MC, 934 (89.5%) had positive HCV RNA, 796 completed anti-HCV therapy, and 715 had sustained virological responses (SVRs). SNP associations were surveyed withgenotypic, allelic, trend, permutation and multivariate analyses. At baseline, higher male sex and MC rates were noted in HCV RNA-positive than RNA-negative patients; higher female sex and positive HCV RNA rates but lower HCV RNA levels were noted in patients with than those without MC. Baseline associations were: HLA II-rs9461776 A allele, IFNL3-rs12979860 T allele, SERPINE1-rs6976053 C allele and MC with HCV RNA positivity; IFNL3-rs12979860 C allele, ARNTL-rs6486122 T allele and HCV RNA positivity with baseline MC. In SVR patients, RETN-rs1423096 C allele and SERPINE1-rs6976053 T allele were associated with 24-week and 10-year post-therapy MC, respectively. Conclusions: HCV RNA, IFNL3-rs12979860 and ARNTL-rs6486122 were associated with baseline MC; RETN-rs1423096 and SERPINE1-rs6976053 were associated with short- and long-term post-therapy MC in SVR patients, respectively. Links with HCV RNA and immune-associated SNPs suggest MC an immune reaction to expel HCV.

## 1. Introduction

Hepatitis C virus (HCV), classified into eight genotypes [1], is a human pathogen responsible for acute and chronic liver disease that chronically infects an estimated 71.1 million individuals worldwide [2]. In addition to hepatic complications including cirrhosis and hepatocellular carcinoma, HCV causes many extrahepatic complications such as mixed cryoglobulinemia [3], hypolipidemia, diabetes and cardiovascular events [4]. Mixed cryoglobulinemia is the most common HCV-associated extrahepatic complications since up to 60% of patients with chronic HCV infection (CHC) have mixed cryoglobulinemia [3]. Several studies had shown the beneficial effects of anti-HCV therapy in curing HCV-associated mixed cryoglobulinemia [3,5]. However, failure to cure mixed cryoglobulinemia or relapse of mixed cryoglobulinemia despite achieving a sustained virological response (SVR) following anti-HCV therapy was not uncommon [3]. Mixed cryoglobulinemia is the prototype of HCV-driven autoimmune disorders [6], affected by the complex interplay among triggering factors and genetic susceptibility [7]. The genetic susceptibility might lead to the persistence of mixed cryoglobulinemia after SVR. Previous studies conducted in Europe and USA had shown the associations of human leukocyte antigen (HLA) class II, neurogenic locus notch homolog protein 4 (NOTCH4) [8] and ATP binding cassette subfamily B member 1 (ABCB1) genes [9] with HCV-associated mixed cryoglobulinemia or cryoglobulinemic vasculitis, while the role of interferon-λ3 (IFNL3) genotype in HCV-associated mixed cryoglobulinemia remained conflicting [10,11]. In addition, the presence of cryoglobulin up-regulates plasminogen activator inhibitor-1 (PAI-1) levels [12], and PAI-1 inhibits HCV replication, which in turn down-regulates PAI-1 expression [13]. The loci at chromosome 7q22.1 close to Serpin Family E Member 1 (SERPINE1) (rs6976053), at chromosome 3p25.2 within peroxisome proliferator-activated receptor gamma (PPARG) (rs11128603) and at chromosome 11p15.2 within aryl hydrocarbon receptor nuclear translocator like (ARNTL) (rs6486122) are all highly associated with PAI-1 levels [14]. Specifically, ARNTL, also known as brain and muscle Arnt-like protein-1 (BMAL1), is a component of the circardian clock [15]. The core clock machinery regulates biosynthesis of nicotinamide phosphoribosyltransferase (NAMPT), a rate-limiting enzyme in mammalian nicotinamide adenine dinucleotide (NAD+) [16]. The genotypes of NAMPT-associated single nucleotide polymorphisms (SNPs), including rs61330082 [17], rs2302559 [18], rs10953502 and rs2058539 [19] were associated with NAMPT profile and might affect cryoglobulin formation through the connection with ARNTL [15] and PAI-1 [14]. In addition, resistin regulates PAI-1 expression via protein kinase B (AKT) phosphorylation [20]. Resistin (RETN)-rs1423096 [21] and RETN-rs1477341 [22] were associated with resistin levels in CHC and in Framingham Offspring studies, respectively. Whether the aforementioned genotypes are associated with HCV-related mixed cryoglobulinemia, particularly in Asia, remained elusive.

Accordingly, we sought to elucidate the genetic profiles associated with HCV-related mixed cryoglobulinemiaby conducting a prospective study to analyze the genetic factors for mixed cryoglobulinemiaof CHC patients in Taiwan, an Asian country where HCV infection is rampant [3,23]. To elucidate the genetic profile for long-term mixed cryoglobulinemia after HCV clearance, the mixed cryoglobulinemia status was followed up in the SVR patientsfor up to 10 years after completion of anti-HCV therapy.

## 2. Materials and Methods

### 2.1. Patients

The study comprised subjects ≥18 years with positive HCV Ab who had been surveyed for serum mixed cryoglobulins. Subjects with human immunodeficiency virus infection, hepatitis B virus infection, hemochromatosis, primary biliary cholangitis, primary sclerosing cholangitis, autoimmune hepatitis or malignancy and recipients of solid organ transplants were excluded. Among the HCV Ab-positive subjects, CHC was defined as detectable HCV RNA by PCR for >24 weeks [3], spontaneous HCV clearance was defined as positive HCV Ab but undetectable HCV RNA without any anti-HCV therapy [24]. Mixed cryoglobulinemia was defined as positive serum mixed cryoglobulins. The diagnosis of mixed cryoglobulinemic syndrome was based on the presence of serum cryoglobulins and positivity for ≥2 of 3 items, including questionnaire, clinical, and laboratory items. In brief, mixed cryoglobulinemic syndrome was defined as circulating cryoglobulin associated with symptoms resulting from purpura, cutaneous ulcers, Raynaud’s phenomenon, arthralgias, sicca syndrome, gastrointestinal vasculitis, neurologic involvement, or renal involvement [3]. HCV-related mixed cryoglobulinemia was defined as the mixed cryoglobulinemia in patients with positive HCV Ab.

### 2.2. Study Design

**Figure 1:**
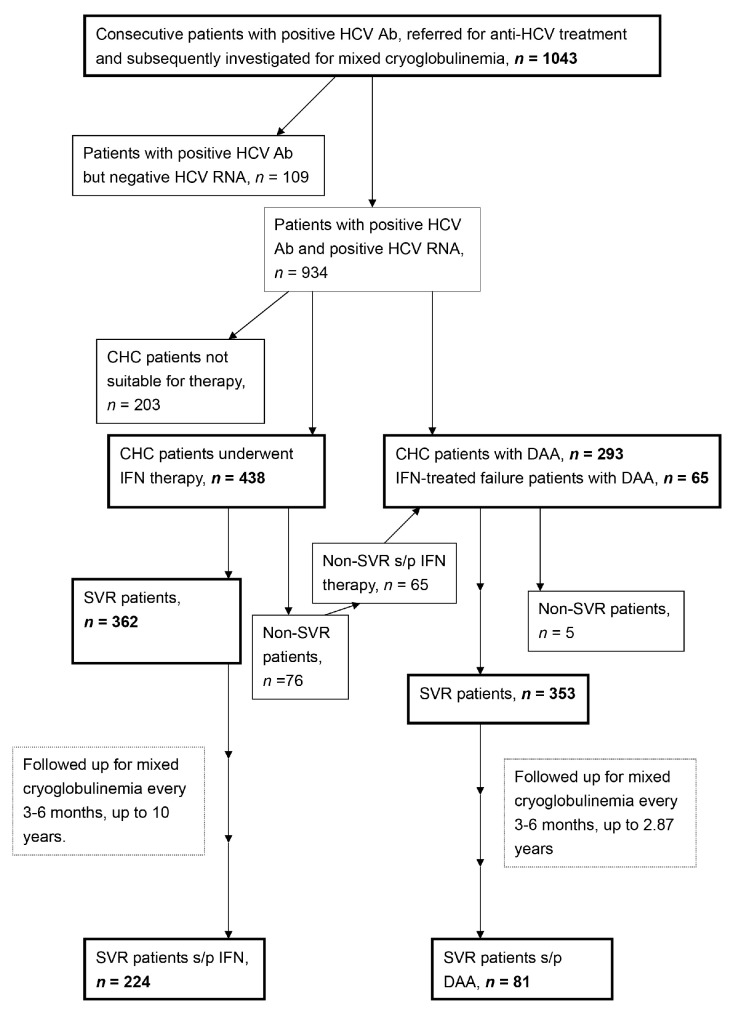
A schematic flow chart of the enrolled patients. HCV Ab: hepatitis C virus antibody; CHC: chronic hepatitis C virus infection; IFN: interferon-based therapy; DAA: direct acting antiviral; s/p: status post; SVR: sustained virological response.

A total of 1043HCV Ab-positive patients were consecutively recruited at a tertiary referral center between January 2010 and May 2019. A schematic flow chart of the enrolled patients was shown in Figure 1. Of 1043, 796 CHC patients had finished the anti-HCV therapy through interferon-based (*n* = 438) [3] or direct-acting antiviral agent (DAA) therapies (*n* = 358) with various combinations (Supplementary Table S1) according to reimburse policy of Bureau of National Health Insurance of the country. Baseline variables including sex, age, mixed cryoglobulinemia, HCV genotypes, levels of HCV RNA and alanine aminotransferase (ALT) were recorded. The SNPs of IFNL3-rs12979860 [3], ABCB1-rs1045642 [9], HLA class II-rs9461776 [8,25], NOTCH4-rs2071286 [25], SERPINE1-rs6976053, ARNTL-rs6486122, PPARG-rs11128603 [14], NAMPT-rs61330082 [17], NAMPT-rs2302559 [18], NAMPT-rs10953502, NAMPT-rs2058539 [19], RETN-rs1423096 and RETN-rs1477341 [21] were assessed using TaqMan SNP Genotyping assays (Applied Biosystems, Waltham, MA, USA) (Supplementary Table S2)or were assessed as described previously [26] (Supplementary Table S3). Biochemical tests were performed at the clinical pathology laboratories of the hospital using routine automated techniques, serum cryoglobulins were measured using the double immunodiffusion method [27]. For the CHC patients who had completed the anti-HCV therapy, an SVR was defined as undetectable levels of HCV RNA at 12 weeks (for DAA therapy) or 24 weeks (for interferon-based therapy) after the completion of therapy. The mixed cryoglobulinemia status was followed in SVR patients every 3–6 months after the completion of therapy.

### 2.3. Statistics

All statistical analyses were performed using the Statistical Package for Social Science (SPSS package version 21, SPSS Inc., Chicago, IL, USA),Statistical Analysis System (SAS version9.4, SAS Institute Inc., Cary, NC, USA), PLINK (version 1.07), HAPLOVIEW (version 4.2), or MassARRAYTyper 4.0 (Sequenom) software. For the genetic analyses, according to our previous studies, population stratification was not indicated because Taiwan’s Han Chinese differ drastically in genotypic information compared with Caucasians but are relatively homogeneous among the three major ethnic subgroups including Minnan, Hakka and Mainlanders [28,29]. SNPs with poor quality were removed using a sequentially exclusive procedure [30]. Genotype association tests were performed using logistic regression analyses with the assumption of an additive or recessive genetic model. Odds ratios and their 95% confidence intervals were calculated. Single-locus association tests were performed in genotype-based, allele-based, and trend-based analyses. Permutation tests based on 100,000 replications were performed to correct for multiple comparisons. Multivariate logistic or Cox regression models were used to assess relationships between various dependent and independent variables by adjusting for all the independent variables with *p* values < 0.1 in univariate analyses. Hosmer-Lemeshow tests were performed to survey the goodness of fit for the multivariate logistic regression models. Statistical significance was defined at the 5% level based on two-tailed tests of the null hypothesis.

### 2.4. Informed Consent

Written informed consent was obtained from each patient. The study protocol conformed to the ethical guidelines of the 1975 Declaration of Helsinki and was approved by the local institutional review board.

## 3. Results

### 3.1. Baseline Characteristics

As shown in Figure 1 and Table 1, of 1043 HCV Ab-positive patients, with a mean age of 57.0 years, 520 (49.9%) were females, 934 (90.4%) had CHC, 109 (10.5%) had spontaneous clearance of HCV, and 589 (56.5%) had baseline mixed cryoglobulinemia. Compared with patients with spontaneous HCV clearance, CHC patients were more frequently male, older, had higher rates of baseline mixed cryoglobulinemia, and genotypes of rs9461776 AA and rs6976053 CC and levels of ALT, and lower rates of rs12979860 CC genotype.

Patients with baseline mixed cryoglobulinemia were more frequently female, older, had higher levels of ALT, rates of HCV positivity, and rs12979860 CC and rs6486122 TT genotypes but lower levels of HCV RNA, than those without mixed cryoglobulinemia (Table 2).

### 3.2. Post-Therapy Mixed Cryoglobulinemia in SVR Patients

At 24 weeks post-therapy, of the 438 patients who had completed a course of interferon-based therapy, mixed cryoglobulinemia was noted in 131 (36.2%) of 362 SVR patients and in 40 (52.6%) of 76 non-SVR patients. Of the 358 patients who had completed a course of DAA therapy, mixed cryoglobulinemia was noted in 85 (24.0%) of 353 SVR patients and 3 (60%) in 5 non-SVR patients. In total, 30.2% (216/715) of the SVR patients had 24-week post-therapy mixed cryoglobulinemia.

Ultimately, for the SVR patients with interferon-based therapy, with a follow-up to 10 years (mean ± standard deviation (SD): 3.92 ± 1.64 years; median: 4.96 years), the long-term post-therapy mixed cryoglobulinemia was noted in 17.0% (38/224) of the interferon-treated SVR patients; for the SVR patients with DAA therapy, with a follow-up to 2.87 years (mean ± SD: 1.19 ± 0.39 years; median: 1.01 years), the long-term post-therapy mixed cryoglobulinemia was noted in 20.9% (17/81) of the DAA-treated SVR patients. In total, regardless of therapeutic regimens, the long-term post-therapy mixed cryoglobulinemia rate was 18.0% (55/305).

### 3.3. Genetic Associations with Baseline HCV RNA

At baseline, all the genotypic, allelic, trend and permutation tests showed, rs12979860, rs6976053, rs2071286, and rs9461776 were associated with baseline HCV RNA positivity (Supplementary Table S4). The univariate and multivariate analysis confirmed, pre-therapy mixed cryoglobulinemia, rs12979860 T allele, rs6976053 C allele, and rs9461776 A allele were positively associated with baseline HCV RNA positivity (Table 3).

### 3.4. Genetic Associations with Baseline Mixed Cryoglobulinemia

At baseline, all the genotypic, allelic, trend and permutation tests showed, rs12979860, and rs6486122 were associated with baseline mixed cryoglobulinemia (Supplementary Table S4). The univariate and multivariate analyses confirmed, HCV RNA positivity, 12979860 C allele, and rs6486122 T allele were positively associated with pre-therapy mixed cryoglobulinemia (Table 4).

Among patients with spontaneous HCV clearance, the rates of rs12979860 CC or rs6486122 TT genotypes were similar between those with and without baseline mixed cryoglobulinemia. By contrast, among CHC patients, a higher rate of rs12979860 CC genotype, and a borderline higher rate of rs6486122 TT genotype were noted in those with than those without baseline mixed cryoglobulinemia (Supplementary Table S5).

None of the investigated SNPs were associated with the presence of cryoglobulinemic vasculitis (Supplementary Table S6).

### 3.5. Genetic Associations with Post-Therapy Mixed Cryoglobulinemia in SVR Patients

Among the SVR patients, the univariate and multivariate analyses confirmed, at 24 weeks post-therapy, pre-therapy mixed cryoglobulinemia and rs1423096 C allele were associated with 24-week post-therapy mixed cryoglobulinemia (Table 5). Up to 10 years post-therapy, 24-week post-therapy mixed cryoglobulinemia and rs6976053 T allele were associated with long-term mixed cryoglobulinemia (Table 6).

## 4. Discussion

That CHC patients had higher mixed cryoglobulinemia rates than patients with spontaneous HCV clearance supports the causal relationship between HCV infection and mixed cryoglobulinemia; while higher positive HCV RNA rates but lower HCV RNA levels in patients with than those without baseline mixed cryoglobulinemia suggested that HCV-related mixed cryoglobulinemia might be stemmed from host’s immune reaction to expel HCV, albeit the expulsion failed in CHC patients. Moreover, that patients with baseline mixed cryoglobulinemia and CHC patients were more frequently female and male, respectively, is consistent with the notions that females are prone to have autoimmune disease [31], have more robust immune response to expel HCV [32] and have a higher spontaneous HCV clearance rate [33].

In addition to baseline mixed cryoglobulinemia, rs6976053 C allele, rs12979860 T allele, and rs9461776 A allele were associated with HCV RNA positivity. Indeed, PAI-1 and HCV have a reciprocal inhibition for each other [13], and rs6976053 C allele is associated with low PAI-1 levels [14], which echoed an efficient HCV replication and thus HCV RNA positivity. rs12979860CC genotype is associated with favorable response to interferon-based therapy and spontaneous HCV clearance [34]. Consistently, CHC patients had a lower rate of rs12979860 CC genotype than patients with spontaneous HCV clearance and rs12979860 T allele is associated with HCV RNA positivity. A broad, vigorous HLA class II-medicated CD4 T cell response favors HCV clearance [35], and both HLA-DRB1*11and HLA-DQB1*03 have been linked to spontaneous HCV clearance [36,37]. That rs9461776 located between HLA-DRB1 and HLA-DQA1 gene segments explains it’s association with HCV RNA positivity.

As mentioned above, rs12979860 T allele was associated with HCV RNA positivity, while HCV RNA positivity and rs12979860 C allele were associated with mixed cryoglobulinemia. Moreover, the association between rs12979860 C allele and mixed cryoglobulinemia was only significant among CHC patients but not among patients with spontaneous HCV clearance. These paradoxical associations support that the immunity is defective to clear HCV in patients carrying the rs12979860 T allele; while among CHC patients carrying rs12979860 C allele, mixed cryoglobulins seemed to result from failure of expelling HCV. Of note, it is a novel finding that rs6486122T allele was associated with baseline mixed cryoglobulinemia. Given that rs6486122 T allele is associated with high PAI-1 levels [14], which inhibit HCV replication [13], it endorsed the concept that emergence of mixed cryoglobulinemia aids to fight for HCV. Although a link of rs2071286 and rs9461776 with HCV-related cryoglobulinemic vasculitis was shown in Europe [8], in our study, neither of the 2 SNPs were associated mixed cryoglobulinemia or cryoglobulinemic vasculitis. Ethnic variation and different study design may account for the discrepancy.

Among SVR patients, pre-therapy mixed cryoglobulinemia and rs1423096 CC genotype were associated with 24-week post-therapy mixed cryoglobulinemia. Our previous study had demonstrated the negative association between rs1423096 C allele and resistin levels in CHC patients [21], and resistin was shown to up-regulate PAI-1 levels [38]. Thus the association of rs1423096 C allele with 24-week post-therapy mixed cryoglobulinemia suggested a link between low PAI-1 levels and 24-week post-therapy mixed cryoglobulinemia. Moreover, the 24-week post-therapy mixed cryoglobulinemia and rs6976053 T allele were associated with long-term post-therapy mixed cryoglobulinemia (up to 10 years), while rs6976053 T allele was linked with high PAI-1 levels [14]. Based on the all the associations: between rs6486122 and pre-therapy mixed cryoglobulinemia, among rs6486122, rs6976053 and PAI-1 levels [14], between rs1423096 C allele and resistin [21], between resistin and PAI-1 levels [38], between rs1423096 C allele and 24-week post-therapy mixed cryoglobulinemia, and between rs6976053T allele and long-term mixed cryoglobulinemia, PAI-1 pathway is crucial for HCV-associated mixed cryoglobulinemia, and the different PAI-1-associated SNPs might have various impacts on the different stage of HCV-associated mixed cryoglobulinemia. Given that PAI-1 is associated with cardiovascular events in SVR patients [39], the link between HCV-associated mixed cryoglobulinemia and cardiovascular risks demand further investigation.

Although the association between presence of HCV-viremia and mixed cryoglobulinemia appears robust, the SNP associations with pre or post-therapy mixed cryoglobulinemia are weaker and might barely reach statistical significance. In many instances, these single-center SNP analyses fail to be reproduced in larger contexts, representing a limitation. Future large-scale prospective studies with HCV-infected patients enrolled from multiple centers are required to verify the links between the SNPs and mixed cryoglobulinemia described herein.

Taken together, pre-therapy mixed cryoglobulinemia, IFNL3-rs12979860, SERPINE1-rs6976053, and HLA class II-rs9461776 were associated with baseline HCV RNA positivity; the presence of HCV RNA, IFNL3-rs12979860, and ARNTL-rs6486122 were associated with pre-therapy mixed cryoglobulinemia; the pre-therapy mixed cryoglobulinemia and RETN-rs1423096 were associated with 24-week post-therapy mixed cryoglobulinemia; and 24-weeks post-therapy mixed cryoglobulinemia and SERPINE1-rs6976053 were associated with 10-year mixed cryoglobulinemia in SVR patients. The presence of HCV-associated mixed cryoglobulinemia likely signify an immune reaction for expelling HCV. The associated SNPs for HCV-associated mixed cryoglobulinemia at various stages mark the vulnearable patients to have mixed cryoglobulinemia, and PAI-1-related pathway might be crucial in HCV-associated mixed cryoglobulinemia.

## Figures and Tables

**Table 1:** Baseline characteristics of 1043 patients with positive HCV Ab.

	GENE	Gentype	Total (*n* = 1043)	CHC (*n* = 934)	Spontaneous HCV Clearance (*n* = 109)	*p* Values (CHC vs. SHC)
Female, *n* (%)			520 (49.9)	449 (48.1)	71 (65.1)	0.001
Age (years old)			57.0 ± 12.95	57.1 ± 12.8	54.3 ± 13.9	0.031
Mixed cryoglobulinemia, *n* (%)			589 (56.5)	550 (58.9)	39 (35.8)	<0.001
Log HCV RNA (logIU/mL)			6.04 ± 1.01	6.04 ± 1.00	NA	
HCV genotype						
Genotype 1, *n* (%)			550 (52.7)	500 (53.5)	NA	
Genotype 2, *n* (%)			300 (28.8)	300 (32.1)	NA	
Others, *n* (%)			134 (12.8)	134 (14.3)	NA	
ALT(U/L)			88.3 ± 99.42	93.6 ± 102.0	42.2 ± 54.3	<0.001
rs12979860, *n* (%)	IFNL3	CC	891 (85.4)	791 (84.7)	100 (91.7)	0.01
rs6486122, *n* (%)	ARNTL	TT	224 (21.4)	207 (22.2)	17 (15.6)	0.09
rs1045642, *n* (%)	ABCB1	GG	422 (40.5)	377 (40.4)	45 (41.3)	0.399
rs9461776, *n* (%)	HLA-II	AA	871 (83.3)	791 (84.7)	80 (73.4)	0.003
rs2071286, *n* (%)	NOTCH4	CC	774 (74.2)	701 (75.1)	73 (67)	0.083
rs6976053, *n* (%)	SERPINE1	CC	292 (27.9)	289 (30.9)	3 (2.8)	<0.001
rs11128603, *n* (%)	PPARG	TT	943 (90.2)	850 (91.0)	93 (85.3)	0.44
rs61330082, *n* (%)	NAMPT	TT	178 (17)	166 (17.8)	12 (11)	0.46
rs10953502, *n* (%)	NAMPT	TT	599 (57.3)	554 (59.3)	45 (41.3)	0.132
rs2302559, *n* (%)	NAMPT	CC	624 (59.7)	576 (61.7)	48 (44)	0.14
rs2058539, *n* (%)	NAMPT	AA	608 (58.2)	562 (60.2)	46 (42)	0.297
rs1423096, *n* (%)	RETN	CC	670 (64.2)	595 (63.7)	75 (69.2)	0.92
rs1477341, *n* (%)	RETN	AA	343 (32.9)	308 (33.0)	35 (32.1)	0.332

HCV: hepatitis C virus; CHC: chronic hepatitis C virus infection; SHC: Spontaneous HCV clearance; ALT: alanine transaminase; IFNL3: interferon-λ 3; ARNTL: aryl hydrocarbon receptor nuclear translocator-like protein 1; ABCB1: ATP binding cassette subfamily B member 1; HLA II: human leukocyte antigen class II; NOTCH4: neurogenic locus notch homolog protein 4; SERPINE1: serpin family E member 1; PPARG: peroxisome proliferator-activated receptor gamma; NAMPT: Nicotinamide phosphoribosyltransferase; RETN: resistin; NA: not assessable.

**Table 2:** Comparison between patients with and without baseline mixed cryoglobulinemia.

	Genotype	Mixed Cryoglobulinemia (+) (*n* = 589)	MixecdCryoglobulinemia (−) (*n* = 454)	*p* Values
Female, *n* (%)		326 (55.3)	194 (42.7)	<0.01
Age (years old)		58.04 ± 12.62	55.21 ± 13.21	<0.01
HCV RNA positivity		550 (93.4)	384 (84.6)	<0.001
Log HCV RNA (logIU/mL)		5.91 ± 1.04	6.23 ± 0.92	<0.001
ALT(U/L)		93.87 ± 98.71	81.08 ± 99.98	0.04
rs12979860, *n* (%)	CC	515 (87.4)	378 (82.8)	0.026
rs6486122, *n* (%)	TT	138 (23.4)	86 (18.9)	0.033
rs1045642, *n* (%)	GG	239 (40.6)	183 (40.3)	0.745
rs9461776, *n* (%)	AA	488 (82.9)	383 (84.4)	0.449
rs2071286, *n* (%)	CC	440 (74.7)	334 (73.6)	0.552
rs6976053, *n* (%)	CC	175 (29.7)	117 (25.7)	0.384
rs11128603, *n* (%)	TT	532 (90.3)	411 (90.5)	0.692
rs61330082, *n* (%)	TT	96 (16.3)	82 (18.1)	0.867
rs10953502, *n* (%)	TT	334 (56.7)	265 (58.4)	0.108
rs2302559, *n* (%)	CC	358 (60.8)	266 (58.6)	0.745
rs2058539, *n* (%)	AA	342 (58.1)	266 (58.6)	0.205
rs1423096, *n* (%)	CC	386 (65.5)	283 (62.3)	0.381
rs1477341, *n* (%)	AA	185 (31.4)	163 (35.9)	0.465

ALT: alanine transaminase; IFNL3: interferon-λ 3; ARNTL: aryl hydrocarbon receptor nuclear translocator-like protein 1;ABCB1: ATP binding cassette subfamily B member 1; HLA II: human leukocyte antigen class II; NOTCH4: neurogenic locus notch homolog protein 4; SERPINE1: serpin family E member 1; PPARG: peroxisome proliferator-activated receptor gamma; NAMPT: Nicotinamide phosphoribosyltransferase; RETN: resistin.

**Table 3:** Genetic associations with baseline HCV RNA positivity.

	Reference Allele/Risk Allele	Univariate Analyses			Multivariate Analysis		
		OR	95% CI OR	*p* Values	OR	95% CI OR	*p* Values
Baseline MC (0,1)		2.618	1.726–3.969	<0.001	2.617	1.679–4.079	<0.001
rs12979860-	C/T	3.72	1.397–9.905	0.009	3.755	1.378–10.235	0.01
rs1045642-	G/A	1.164	0.856–1.582	0.332			
rs9461776	G/A	1.751	1.125–2.276	0.013	1.783	1.106–2.876	0.018
rs2071286	C/T	0.656	0.441–0.974	0.037	0.711	0.468–1.08	0.11
rs6976053	T/C	2.871	2.045–4.131	<0.001	2.737	1.93–3.881	<0.001
rs6486122	C/T	0.942	0.725–1.224	0.657			
rs11128603-	G/A	0.744	0.29–1.911	0.539			
rs61330082-	C/T	0.9	0.603–1.343	0.605			
rs2302559-	C/T	1.867	0.859–4.056	0.115			
rs10953502-	C/T	1.243	0.591–2.616	0.567			
rs2058539-	C/T	0.519	0.232–1.162	0.111			
rs1423096-	C/T	1.182	0.48–2.906	0.716			
rs1477341-	A/T	0.728	0.191–2.772	0.642			

MC: mixed cryoglobulinemia; IFNL3: interferon λ3; ABCB1: ATP binding cassette subfamily B member 1; HLA II: Human leucocyte antigen class II; NOTCH4: neurogenic locus notch homolog protein 4; SERPINE1: Serpin Family E Member 1; ARNTL: aryl hydrocarbon receptor nuclear translocator like; PPARG: peroxisome proliferator-activated receptor gamma; NAMP: Nicotinamide phosphoribosyltransferase; RETN: resistin; OR: odds ratio. *p* value for Hosmer-Lemeshow test: 0.963.

**Table 4:** Genetic associations with pre-therapy mixed cryoglobulinemia.

	Reference Allele/Risk Allele	Univariate Analyses			Multivariate Analysis		
		OR	95% CI OR	*p* Values	OR	95% CI OR	*p* Values
HCV RNA positivity		2.618	1.726–3.969	<0.001	2.556	1.673–3.915	<0.001
rs12979860	T/C	1.142	1.01–1.974	0.044	1.531	1.087–2.056	0.015
rs1045642	G/A	1.016	0.835–1.236	0.877			
rs9461776	G/A	0.917	0.648–1.298	0.624			
rs2071286	C/T	0.894	0.672–1.188	0.44			
rs6976053	T/C	0.907	0.776–1.062	0.226			
rs6486122	C/T	1.19	1.002–1.414	0.048	1.191	1.000–1.419	0.049
rs11128603	G/A	0.943	0.547–1.624	0.832			
rs61330082	C/T	0.937	0.751–1.168	0.56			
rs2302559	C/T	0.901	0.653–1.244	0.527			
rs10953502	C/T	1.293	0.89–1.877	0.177			
rs2058539	C/T	0.796	0.56–1.113	0.205			
rs1423096	C/T	0.854	0.656–1.112	0.241			
rs1477341	A/T	0.961	0.73–1.264	0.774			

IFNL3: interferon λ3; ABCB1: ATP binding cassette subfamily B member 1; HLA II: Human leucocyte antigen class II; NOTCH4: neurogenic locus notch homolog protein 4; SERPINE1: Serpin Family E Member 1; ARNTL: aryl hydrocarbon receptor nuclear translocator like; PPARG: peroxisome proliferator-activated receptor gamma; NAMP: Nicotinamide phosphoribosyltransferase; RETN: resistin; OR: odds ratio. *p* value for Hosmer-Lemeshow test:0.896.

**Table 5:** Genetic associations with 24-week post-therapy mixed cryoglobulinemia in SVR patients.

	Reference Allele/Risk Allele	Univariate Analyses			Multivariate Analysis		
		OR	95% CI OR	*p* Values	OR	95% CI OR	*p* Values
Pre-therapy mixed cryoglobulinemia		3.143	2.16–4.575	<0.001	3.113	1.895–5.116	<0.001
rs12979860	T/C	1.283	0.763–2.159	0.348			
rs1045642	G/A	1.031	0.774–1.372	0.835			
rs9461776	G/A	1.068	0.649–1.757	0.797			
rs2071286	C/T	1.035	0.678–1.58	0.874			
rs6976053	C/T	0.933	0.746–1.166	0.541			
rs6486122	C/T	1.024	0.802–1.308	0.85			
rs11128603	G/A	1.414	0.585–3.42	0.442			
rs61330082	C/T	0.857	0.64–1.147	0.299			
rs2302559	C/T	1.142	0.734–1.776	0.557			
rs10953502	C/T	1.013	0.588–1.743	0.964			
rs2058539	C/T	0.989	0.601–1.627	0.965			
rs1423096	C/T	0.652	0.45–0.944	0.023	0.677	0.46–0.995	0.047
rs1477341	A/T	0.794	0.589–1.072	0.132			

SVR: sustained virological response; IFNL3: interferon λ3; ABCB1: ATP binding cassette subfamily B member 1; HLA II: Human leucocyte antigen class II; NOTCH4: neurogenic locus notch homolog protein 4; SERPINE1: Serpin Family E Member 1; ARNTL: aryl hydrocarbon receptor nuclear translocator like; PPARG: peroxisome proliferator-activated receptor gamma; NAMP: Nicotinamide phosphoribosyltransferase; RETN: resistin; OR: odds ratio; CI; confidence interval.

**Table 6:** Genetic associations with long-term post-therapy mixed cryoglobulinemia in SVR patients.

	Reference Allele/Risk Allele	Univariate Analyses			Multivariate Analysis		
		HR	95% CI HR	*p* Values	HR	95% CI HR	*p* Values
24-week post-therapy mixed cryoglobulinemia (0,1)		3.145	1.997–4.952	<0.001	2.829	1.779–4.497	<0.001
rs12979860	T/C	1.76	0.911–3.403	0.093	1.888	0.867–4.114	0.11
rs1045642	G/A	0.906	0.669–1.229	0.526			
rs9461776	G/A	2.121	0.985–4.566	0.055	1.982	0.927–4.235	0.078
rs2071286	C/T	0.971	0.605–1.558	0.903			
rs6976053	C/T	1.313	1.023–1.694	0.032	1.351	1.046–1.746	0.021
rs6486122	C/T	0.861	0.661–1.122	0.268			
rs11128603	G/A	0.719	0.332–1.56	0.404			
rs61330082	C/T	1.144	0.839–1.56	0.396			
rs2302559	C/T	0.927	0.559–1.538	0.769			
rs10953502	C/T	0.825	0.412–1.653	0.588			
rs2058539	C/T	1.194	0.61–2.34	0.605			
rs1423096	C/T	0.798	0.538–1.183	0.261			
rs1477341	A/T	1.141	0.787–1.654	0.487			

SVR: sustained virological response; IFNL3: interferon λ3; ABCB1: ATP binding cassette subfamily B member 1; HLA II: Human leucocyte antigen class II; NOTCH4: neurogenic locus notch homolog protein 4; SERPINE1: Serpin Family E Member 1; ARNTL: aryl hydrocarbon receptor nuclear translocator like; PPARG: peroxisome proliferator-activated receptor gamma; NAMP: Nicotinamide phosphoribosyltransferase; RETN: resistin; HR: hazard ratio; CI; confidence interval.

## Data Availability

The datasets used and/or analysed during the current study are available from the corresponding author on reasonable request.

## Supplementary Materials

The following are available online at https://www.mdpi.com/1999-4915/13/3/464/s1, Table S1: Various DAA combinations used in the study, Table S2: The 13 single-nucleotide polymorphisms evaluated in the study, Table S3: Primer sequences used in the single-nucleotide polymorphisms of NAMPT-rs61330082, Table S4: Genetic analyses for various single-nucleotide polymorphisms with HCV RNA and mixed cryoglobullinemia, Table S5: IFNL3-rs12978960 variant distributions among various status, Table S6: Genetic associations with pre-therapy cyoglobulinemic vasculitis.

## References

[1] Borgia S.M., Hedskog C., Parhy B., Hyland R.H., Stamm L.M., Brainard D.M., Subramanian M.G., McHutchison J.G., Mo H., Svarovskaia E. (2018). Identification of a Novel Hepatitis C Virus Genotype from Punjab, India: Expanding Classification of Hepatitis C Virus into 8 Genotypes. J. Infect. Dis..

[2] Spearman C.W., Dusheiko G.M., Hellard M., Sonderup M. (2019). Hepatitis C. Lancet.

[3] Cheng Y.T., Cheng J.S., Lin C.H., Chen T.H., Lee K.C., Chang M.L. (2020). Rheumatoid factor and immunoglobulin M mark hepatitis C-associated mixed cryoglobulinaemia: An 8-year prospective study. Clin. Microbiol. Infect..

[4] Chang M.L. (2016). Metabolic alterations and hepatitis C: From bench to bedside. World J. Gastroenterol..

[5] Gragnani L., Visentini M., Fognani E., Urraro T., De Santis A., Petraccia L., Perez M., Ceccotti G., Colantuono S., Mitrevski M. (2016). Prospective study of guideline-tailored therapy with direct-acting antivirals for hepatitis C virus-associated mixed cryoglobulinemia. Hepatology.

[6] Zignego A.L., Gragnani L., Piluso A., Sebastiani M., Giuggioli D., Fallahi P., Antonelli A., Ferri C. (2015). Virus-driven autoimmunity and lymphoproliferation: The example of HCV infection.Virus-driven autoimmunity and lymphoproliferation: The example of HCV infection. Expert Rev. Clin. Immunol..

[7] Praprotnik S., Sodin-Semrl S., Tomsic M., Shoenfeld Y. (2008). The curiously suspicious: Infectious disease may ameliorate an ongoing autoimmune destruction in systemic lupus erythematosus patients. J. Autoimmun..

[8] Zignego A.L., Wojcik G.L., Cacoub P., Visentini M., Casato M., Mangia A., Latanich R., Charles E.D., Gragnani L., Terrier B. (2014). Genome-wide association study of hepatitis C virus—and cryoglobulin-related vasculitis. Genes Immun..

[9] Cusato J., Boglione L., De Nicolò A., Cardellino C.S., Carcieri C., Cariti G., Di Perri G., D’Avolio A. (2017). Pharmacogenetic analysis of hepatitis C virus related mixed cryoglobulinemia. Pharmacogenomics.

[10] Piluso A., Giannini C., Fognani E., Gragnani L., Caini P., Monti M., Petrarca A., Ranieri J., Urraro T., Triboli E. (2013). Value of IL28B genotyping in patients with HCV-related mixed cryoglobulinemia: Results of a large, prospective study. J. Viral. Hepat..

[11] Boglione L., Cusato J., Allegra S., Cariti G., Di Perri G., D’avolio A. (2015). Role of IL28B genotyping in patients with hepatitis C virus-associated mixed cryoglobulinemia and response to PEG-IFN and ribavirin treatment. Arch. Virol..

[12] Taneda S., Hudkins K.L., Mühlfeld A.S., Kowalewska J., Pippin J.W., Shankland S.J., Alpers C.E. (2008). Protease nexin-1, tPA, and PAI-1 are upregulated in cryoglobulinemic membranoproliferative glomerulonephritis. J. Am. Soc. Nephrol..

[13] Yang C.H., Li H.C., Ku T.S., Wu P.C., Yeh Y.J., Cheng J.C., Lin T.Y., Lo S.Y. (2017). Hepatitis C virus down-regulates SERPINE1/PAI-1 expression to facilitate its replication. J. Gen. Virol..

[14] Huang J., Sabater-Lleal M., Asselbergs F.W., Tregouet D., Shin S.Y., Ding J., Baumert J., Oudot-Mellakh T., Folkersen L., Johnson A.D. (2012). Genome-wide association study for circulating levels of PAI-1 provides novel insights into its regulation. Blood.

[15] Shimba S., Ishii N., Ohta Y., Ohno T., Watabe Y., Hayashi M., Wada T., Aoyagi T., Tezuka M. (2005). Brain and muscle Arnt-like protein-1 (BMAL1), a component of the molecular clock, regulates adipogenesis. Proc. Natl. Acad. Sci. USA.

[16] Ramsey K.M., Yoshino J., Brace C.S., Abrassart D., Kobayashi Y., Marcheva B., Hong H.K., Chong J.L., Buhr E.D., Lee C. (2009). Circadian clock feedback cycle through NAMPT-mediated NAD+ biosynthesis. Science.

[17] Ooi D.S., Ong S.G., Heng C.K., Loke K.Y., Lee Y.S. (2016). In-vitro function of upstream visfatin polymorphisms that are associated with adverse cardiometabolic parameters in obese children. BMC Genom..

[18] Stastny J., Bienertova-Vasku J., Tomandl J., Tomandlova M., Zlamal F., Forejt M., Splichal Z., Vasku A. (2013). Association of genetic variability in selected regions in visfatin (NAMPT) gene with anthropometric parameters and dietary composition in obese and non-obese Central-European population. Diabetes MetabSyndr..

[19] Jian W.X., Luo T.H., Gu Y.Y., Zhang H.L., Zheng S., Dai M., Han J.F., Zhao Y., Li G., Luo M. (2006). The visfatin gene is associated with glucose and lipid metabolism in a Chinese population. Diabet. Med..

[20] Ikeda Y., Tsuchiya H., Hama S., Kajimoto K., Kogure K. (2014). Resistin regulates the expression of plasminogen activator inhibitor-1 in 3T3-L1 adipocytes. Biochem. Biophys. Res. Commun..

[21] Chang M.L., Liang K.H., Ku C.L., Lo C.C., Cheng Y.T., Hsu C.M., Yeh C.T., Chiu C.T. (2016). Resistin reinforces interferon λ-3 to eliminate hepatitis C virus with fine-tuning from RETN single-nucleotide polymorphisms. Sci. Rep..

[22] Hivert M.F., Manning A.K., McAteer J.B., Dupuis J., Fox C.S., Cupples L.A., Meigs J.B., Florez J.C. (2009). Association of variants in RETN with plasma resistin levels and diabetes-related traits in the Framingham Offspring Study. Diabetes.

[23] Hu J.H., Chen M.Y., Yeh C.T., Lin H.S., Lin M.S., Huang T.J., Chang M.L. (2016). Sexual Dimorphic Metabolic Alterations in Hepatitis C Virus-infected Patients: A Community-Based Study in a Hepatitis B/Hepatitis C Virus Hyperendemic Area. Med. Baltim..

[24] Lee K.C., Cheng Y.T., Lin C.Y., Kuo C.J., Chien R.N., Yeh C.T., Chang M.L. (2020). Impact of mixed cryoglobulinemia on patients with spontaneous hepatitis C virus clearance: A 13-year prospective cohort study. Eur. J. Clin. Investig..

[25] Gragnani L., Fognani E., De Re V., Libra M., Garozzo A., Caini P., Cerretelli G., Giovannelli A., Lorini S., Monti M. (2017). Notch4 and mhc class II polymorphisms are associated with hcv-related benign and malignant lymphoproliferative diseases. Oncotarget.

[26] Zhang K., Zhou B., Zhang P., Zhang Z., Chen P., Pu Y., Song Y., Zhang L. (2014). Genetic variants in NAMPT predict bladder cancer risk and prognosis in individuals from southwest Chinese Han group. Tumour Biol..

[27] Motyckova G., Murali M. (2011). Laboratory testing for cryoglobulins. Am. J. Hematol..

[28] Hsieh A.R., Chang S.W., Chen P.L., Chu C.C., Hsiao C.L., Yang W.S., Chang C.C., Wu J.Y., Chen Y.T., Chang T.C. (2014). Predicting HLA genotypes using unphased and flanking single-nucleotide polymorphisms in Han Chinese population. BMC Genom..

[29] Yang H.C., Lin C.H., Hsu C.L., Hung S.I., Wu J.Y., Pan W.H., Chen Y.T., Fann C.S. (2006). A comparison of major histocompatibility complex SNPs in Han Chinese residing in Taiwan and Caucasians. J. Biomed. Sci..

[30] Benjamini Y., Hochberg Y. (1995). Controlling the false discovery rate: A practical and powerful approach to multiple testing. J. R. Stat. Soc. Ser. B Methodol..

[31] Fairweather D., Rose N.R. (2004). Women and autoimmune diseases. Emerg. Infect. Dis..

[32] Klein S.L. (2012). Sex influences immune responses to viruses, and efficacy of prophylaxis and treatments for viral diseases. Bioessays.

[33] Grebely J., Page K., Sacks-Davis R., van der Loeff M.S., Rice T.M., Bruneau J., Morris M.D., Hajarizadeh B., Amin J., Cox A.L. (2014). The effects of female sex, viral genotype, and IL28B genotype on spontaneous clearance of acute hepatitis C virus infection. Hepatology.

[34] Ge D., Fellay J., Thompson A.J., Simon J.S., Shianna K.V., Urban T.J., Heinzen E.L., Qiu P., Bertelsen A.H., Muir A.J. (2009). Genetic variationin IL28B predicts hepatitis Ctreatment-induced viral clearance. Nature.

[35] Thio C.L., Thomas D.L., Goedert J.J., Vlahov D., Nelson K.E., Hilgartner M.W., O’Brien S.J., Karacki P., Marti D., Astemborski J. (2001). Racial differences in HLA class II associations with hepatitis C virus outcomes. J. Infect. Dis..

[36] Honegger J.R., Tedesco D., Kohout J.A., Prasad M.R., Price A.A., Lindquist T., Ohmer S., Moore-Clingenpeel M., Grakoui A., Walker C.M. (2016). Influence of IFNL3 and HLA-DPB1 genotype on postpartum control of hepatitis C virus replication and T-cell recovery. Proc. Natl. Acad. Sci. USA.

[37] Harris R.A., Sugimoto K., Kaplan D.E., Ikeda F., Kamoun M., Chang K.M. (2008). Human leukocyte antigen class II associations with hepatitis C virus clearance and virus-specific CD4 T cell response among Caucasians and African Americans. Hepatology.

[38] Ding Y., Li X. (2019). Resistin Promotes Thrombosis in Rats with Deep Vein Thrombosis via Up-Regulating MMP-2, MMP-9, and PAI-1. Clin. Lab..

[39] Chang M.L., Lin Y.S., Pao L.H., Huang H.C., Chiu C.T. (2017). Link between plasminogen activator inhibitor-1 and cardiovascular risk in chronic hepatitis C after viral clearance. Sci. Rep..

