# Supplementary material for: Genetic Association of Hepatitis C-Related Mixed Cryoglobulinemia: A 10-Year Prospective Study of Asians Treated with Antivirals
Source: Viruses. 2021 Mar 11;13(3):464. doi: 10.3390/v13030464 (PMC7998980; doi:10.3390/v13030464)
Supplement: Supplementary file 1 [file viruses-13-00464-s001.pdf]

Supplementary Table 1. Various DAA combinations used in the study.

Genotype 1		Genotype 2		Genotype 3		Genotype 4		Genotype 5		Genotype 6	
DAA	D(W)	DAA	D(W)	DAA	D(W)	DAA	D(W)	DAA	D(W)	DAA	D(W)
Asunaprevir (100 mg, bid) with Daclatasvir (60 mg, qd)	24	Sovaldi (Sofosbuvir 400 mg qd) with Ribavirin	12								
Viekirax (Ombitasvir 12.5mg/Paritaprevir 75mg/Ritonavir 50 mg qd) with Exviera (250 mg bid)	12-24										
Zepatier (Elbasvir 50 mg/Grazoprevir 100 mg, qd)	12-16										
Harvoni (Ledipasvir 90 mg /Sofosbuvir 400 mg qd) with/without Ribavinin (800-1400 mg/day, in two divided doses	12	Harvoni (Ledipasvir 90 mg /Sofosbuvir 400 mg qd) with/without Ribavinin (800-1400 mg/day, in two divided doses	12			Harvoni (Ledipasvir 90 mg /Sofosbuvir 400 mg qd) with/without Ribavinin (800-1400 mg/day, in two divided	12	Harvoni (Ledipasvir 90 mg /Sofosbuvir 400 mg qd) with/without Ribavinin (800-1400 mg/day, in two divided	12	Harvoni (Ledipasvir 90 mg /Sofosbuvir 400 mg qd) with/without Ribavinin (800-1400 mg/day, in two divided	12
Mavyret (glecaprevir 100mg/pibrentasvir 40mg, 3# qd)	8-16	Mavyret (glecaprevir 100mg/pibrentasvir 40mg, 3# qd)	8-12	Mavyret (glecaprevir 100mg/pibrentasvir 40mg, 3# qd)	8-16	Mavyret (glecaprevir 100mg/pibrentasvir 40mg, 3# qd)	8-12	Mavyret (glecaprevir 100mg/pibrentasvir 40mg, 3# qd)	8-12	Mavyret (glecaprevir 100mg/pibrentasvir 40mg, 3# qd)	8-12
Epclusa (sofosbuvir 400	12	Epclusa (sofosbuvir 400	12	Epclusa (sofosbuvir 400	12	Epclusa (sofosbuvir 400	12	Epclusa (sofosbuvir 400	12	Epclusa (sofosbuvir 400	12

mg/velpatasvir 100 mg qd) with or without Ribavirin	mg/velpatasvir 100 mg qd) with or without Ribavirin	mg/velpatasvir 100 mg qd) with or without Ribavirin	mg/velpatasvir 100 mg qd) with or without Ribavirin	mg/velpatasvir 100 mg qd) with or without Ribavirin	mg/velpatasvir 100 mg qd) with or without Ribavirin

DAA: direct-acting antiviral agent; D: duration: W: weeks

Supplementary Table 2. The 13 single-nucleotide polymorphisms evaluated in the study.

Gene	SNP ID	ID number of TaqMan assays	Chromosome	Location	Risk allele	MAF	Allele 2
IFNL3	rs12979860	C___7820464_10	19q13.2	39248147	T	0.07532	C
ABCB1	rs1045642	C___7586657_20	7q21.12	87138645	A	0.3571	G
HLA II	rs9461776	C__30460277_20	6p21.32	32575735	G	0.07171	A
NOTCH4	rs2071286	C__15861180_10	6p21.32	32179896	T	0.1226	C
ACHE	rs6976053	C__32067460_10	7q22.1	100512119	C	0.4056	T
ARNTL	rs6486122	C___2160490_10	11p15.2	13361524	T	0.3909	C
PPARG	rs11128603	NA	3p25.2	12385828	G	0.03038	A
NAMPT	rs61330082	NA	7q22.3	105926865	T	0.4892	C
NAMPT	rs2302559	C___2673293_10	7q22.3	105903904	T	0.1097	C
NAMPT	rs10953502	C__31761492_10	7q22.3	105892431	C	0.1454	T
NAMPT	rs2058539	C__11613113_10	7q22.3	105916637	C	0.1097	A
RETN	rs1423096	C__1394117_20	19p13.2	7674290	C	0.28332	T
RETN	rs1477341	C__8351534_10	19p13.2	7671912	A	0.47552	T

IFNL3: interferon λ3; ABCB1: ATP-binding cassette sub-family B member 1; HLA II: human leukocyte antigen class II; NOTCH4: neurogenic locus notch homolog protein 4; SERPINE1: Serpin family E member 1; ARNTL: aryl hydrocarbon receptor nuclear translocator-like protein 1; PPARG: peroxisome proliferator-activated receptor gamma; NAMP: Nicotinamide phosphoribosyltransferase; RETN: resistin; NA: not assessible; MAF: minor allele frequency.

Supplementary Table 3. Primer sequences used in the single-nucleotide polymorphisms of NAMPT-rs61330082.

Genes	Primers	Sequences (5'->3')
NAMPT	NAMPT-F	5′- TGTTTCAAACCTCGTT GCTG -3′
	NAMPT-R	5′- GAGGCATGGCTGAGACTTCTA -3′

ScrFI for rs61330082—Allele C is cuttable, yielding two fragments of 65 and 138 bp; allele T is uncuttable, and the fragment is still 203 bp.

Supplementary Table 4. Genetic analyses for various single-nucleotide polymorphisms with HCV RNA and mixed cryoglobullinemia.

SNPs	A1	A2	Y	allele1	allele2	geno11	geno12	geno22	Geno *p*	Trend *p*	Allelic *p*	Dom *p*	Rec *p*	Add *p*	OR	95% CI OR	Permu *p*
rs11128603	G	A	RNA	0.03038	0.96962	0	0.060757	0.939243	0.8255	0.671	0.8283	0.8255	1	0.6715	1.225	0.4789-3.135	0.6712
rs11128603	G	A	MC	0.03038	0.96962	0	0.060757	0.939243	0.7901	0.6913	0.7933	0.7901	1	0.6914	1.112	0.6573-1.883	0.6998
rs2071286	T	C	RNA	0.1226	0.8774	0.013903	0.217478	0.76862	0.01202	0.02822	0.03317	0.139	0.0096	0.02956	0.6448	0.4343-0.957	0.02862
rs2071286	T	C	MC	0.1226	0.8774	0.013903	0.217478	0.76862	0.7903	0.5518	0.5839	0.6513	0.7873	0.5519	0.9216	0.7041-1.206	0.5525
rs9461776	G	A	RNA	0.07171	0.92829	0.010956	0.121514	0.86753	0.007141	0.006055	0.006726	0.003383	1	0.007159	0.5492	0.3548-0.85	0.00623
rs9461776	G	A	MC	0.07171	0.92829	0.010956	0.121514	0.86753	0.6394	0.448	0.4853	0.3987	1	0.4484	1.137	0.816-1.584	0.4481
rs1045642	A	G	RNA	0.3571	0.6429	0.128684	0.456778	0.414538	0.4185	0.3394	0.36	0.7528	0.2161	0.3399	1.16	0.8554-1.573	0.3401
rs1045642	A	G	MC	0.3571	0.6429	0.128684	0.456778	0.414538	0.631	0.7451	0.744	0.898	0.3968	0.7451	0.9702	0.8083-1.164	0.7454
rs6976053	C	T	RNA	0.4056	0.5944	0.287119	0.236971	0.47591	$1.04X10^{-12}$	$9.44x10^{-11}$	$4.79x10^{-17}$	$3.97x10^{-8}$	$1.73x10^{-12}$	$2.64x10^{-09}$	2.767	1.979-3.868	0.00001
rs6976053	C	T	MC	0.4056	0.5944	0.287119	0.236971	0.47591	0.3214	0.3836	0.2948	0.8002	0.1846	0.3837	1.067	0.9222-1.234	0.3833
rs10953502	C	T	RNA	0.1454	0.8546	0.061776	0.16731	0.770914	0.1107	0.06614	0.03219	0.179	0.068	0.07314	1.832	0.9448-3.551	0.06896
rs10953502	C	T	MC	0.1454	0.8546	0.061776	0.16731	0.770914	0.283	0.1125	0.06938	0.1214	0.3663	0.1136	1.227	0.9523-1.582	0.1126
rs2302559	T	C	RNA	0.1131	0.8869	0.015171	0.195954	0.788875	0.1058	0.1044	0.1152	0.08215	0.5746	0.1102	1.885	0.8659-4.104	0.1059
rs2302559	T	C	MC	0.1131	0.8869	0.015171	0.195954	0.788875	0.9351	0.7442	0.749	0.7252	1	0.7442	0.9497	0.6967-1.295	0.7481
rs2058539	C	A	RNA	0.1097	0.8903	0.003871	0.211613	0.784516	0.3249	0.1197	0.1495	0.165	1	0.1253	1.877	0.8392-4.198	0.122
rs2058539	C	A	MC	0.1097	0.8903	0.003871	0.211613	0.784516	0.4868	0.2046	0.2499	0.2173	1	0.2052	1.247	0.8862-1.754	0.2048
rs61330082	T	C	RNA	0.4892	0.5108	0.192641	0.593074	0.214286	0.4819	0.4915	0.5827	0.2723	1	0.4917	0.8695	0.5836-1.295	0.4996
rs61330082	T	C	MC	0.4892	0.5108	0.192641	0.593074	0.214286	0.4608	0.8664	0.888	0.5713	0.3998	0.8664	0.9826	0.8011-1.205	0.8678
rs6486122	T	C	RNA	0.3909	0.6091	0.219178	0.343444	0.437378	0.009836	0.7211	0.708	0.0948	0.1692	0.7211	0.954	0.7367-1.235	0.7158
rs6486122	T	C	MC	0.3909	0.6091	0.219178	0.343444	0.437378	0.1045	0.03313	0.01738	0.05637	0.09375	0.03337	1.189	1.014-1.395	0.03284
rs12979860	T	C	RNA	0.07532	0.92468	0.016521	0.11759	0.865889	0.005611	0.00264	0.000428	0.001238	0.4033	0.005809	3.98	1.492-10.62	0.00428
rs12979860	T	C	MC	0.07532	0.92468	0.016521	0.11759	0.865889	0.06041	0.0215	0.01452	0.0428	0.08676	0.0226	0.6996	0.5146-0.951	0.0203
rs1423096	C	T	RNA	0.28332	0.71668	0.087195	0.272885	0.639920	0.889963	0.7188	0.83269	0.71832	0.99321	0.70869	1.180	0.478-2.908	0.8956
rs1423096	C	T	MC	0.28332	0.71668	0.087195	0.272885	0.639920	0.1932	0.1953	0.1023	0.3852	0.22365	0.198	0.829	0.625-1.099	0.2369
rs1477341	A	T	RNA	0.47552	0.52448	0.257660	0.412359	0.329981	0.632123	0.63889	0.77236	0.2358	0.8236520	0.56321	0.7269	0.192-2.751	0.4463
rs1477341	A	T	MC	0.47552	0.52448	0.257660	0.412359	0.329981	0.7472	0.8659	0.7236	0.5689	0.7963	0.4332	0.9552	0.7222-1.2638	0.7896

A1: allele 1 frequency; A2: allele 2 frequency; Y: dependent factor; RNA: HCV RNA; MC: mixed cryoglobulinemia; geno11: genotype 11 frequency; geno12: genotype 12 frequency; geno 22: genotype 22 frequency; Geno *p*: *p* values of genotypic test; Trend *p*: *p* values of trend test; Allelic *p*: *p* values of allelic test; Dom *p*: *p* values of associated test (genetic model=dominant); Rec *p*: *p* values of associated test (genetic model=recessive); Add *p*: *p* values of logistic regression(genetic model=additive) for qualitative trait; OR: odds ration; CI: confidence interval; Permu *p*: *p* values of permutation test (N=100000).

Supplementary Table 5. IFNL3-rs12978960 variant distributions among various status.

	CHC (n=934)			Spontaneous HCV clearance (n=109)		
	MC (0) (n=384)	MC (1) (n=550)	*p* values	MC (0) (n=70)	MC (1) (n=39)	*p* values
IFNL3-rs12979860 CC genotype, n (%)	314 (81.8)	477 (86.7)	0.011	67 (95.7)	38 (97.4)	0.518
ARNTL-rs6486122 TT genotype, n (%)	75 (19.5)	132 (24)	0.059	11 (15.7)	6 (15.4)	0.191

CHC: chronic hepatitis C virus infection; MC: mixed cryoglobulinemia

Supplementary Table 6. Genetic associations with pre-therapy cyoglobulinemic vasculitis.

	Univariate analyses		
	OR	95% CI OR	*p* values
HCV RNA positivity	$3.53X10^7$	0.00-	0.996
IFNL3-rs12979860 TT, TC, CC genotype (0, 1, 2)	0.762	0.292-1.993	0.58
ABCB1-rs1045642 GG, GA, AA genotype (0, 1, 2)	1.337	0.71-2.516	0.368
HLA II-rs9461776 GG, GA, AA genotype (0, 1, 2)	1.437	0.359-5.757	0.608
NOTCH4-rs2071286 CC, CT, TT genotype (0, 1, 2)	0.368	0.088-1.544	0.172
SERPINE1-rs6976053 TT, TC, CC genotype (0, 1, 2)	1.091	0.644-1.848	0.746
ARNTL-rs6486122 CC, CT, TT genotype (0, 1, 2)	0.948	0.535-1.679	0.854
PPARG-rs11128603 GG, GA, AA genotype (0, 1, 2)	0.574	0.13-2.533	0.464
NAMPT-rs61330082 CC, CT, TT genotype (0, 1, 2)	0.821	0.409-1.648	0.579
NAMPT-rs2302559 CC, CT, TT genotype (0, 1, 2)	0.922	0.327-2.599	0.878
NAMPT-rs10953502 CC, CT, TT genotype (0, 1, 2)	1.242	0.608-2.538	0.553
NAMPT-rs2058539 CC, CT, TT genotype (0, 1, 2)	0.495	0.2-1.222	0.127
RETN-rs1423096 CC, CT, TT genotype (0, 1, 2)	1.322	0.708-2.468	0.381
RETN-rs1477341 AA, AT, AT genotype (0, 1, 2)	3.574	0.842-15.178	0.084

IFNL3: interferon λ3; ABCB1: ATP binding cassette subfamily B member 1; HLA II: Human leucocyte antigen class II; NOTCH4: neurogenic locus notch homolog protein 4; SERPINE1: Serpin Family E Member 1; ARNTL: aryl hydrocarbon receptor nuclear translocator like; PPARG: peroxisome proliferator-activated receptor gamma; NAMP: Nicotinamide phosphoribosyltransferase; OR: odds ratio.
